# Evaluating well-being and psychosocial risks in academia: Is management the “forgotten phase”?

**DOI:** 10.3389/fpsyg.2024.1349589

**Published:** 2024-07-03

**Authors:** Andreina Bruno, Giuseppina Dell’Aversana, Silvia Gilardi

**Affiliations:** ^1^Department of Education Sciences, Università degli studi di Genova, Genoa, Italy; ^2^Department of Psychology, Università degli studi Milano-Bicocca, Milan, Italy; ^3^Department of Social and Political Sciences, Università degli studi di Milano, Milan, Italy

**Keywords:** healthy university, work-related stress, implementation gap, psychosocial work environment, psychosocial risks, higher education, evaluation

## Abstract

In recent years, there has been a noticeable increase in attention towards promoting well-being within academic settings. In the specific context of academia, a critical issue is understanding whether the current practices for assessing and managing well-being can bridge the implementation gap and increase opportunities for creating healthy academic conditions. The paper explores the practices adopted for assessing and managing work-related stress (WRS) risks in Italian academia by referring to data from a group of Italian universities of the QoL@Work network (Quality of Life at Work in academia). The aim is to improve understanding of the factors that influence the realization of a WRS risk assessment-management pathway and how they may facilitate or hinder the transition from assessment to the implementation of interventions in the academic context. The results suggest that the assessment-management pathway should prioritize the creation of organizational scaffolding to support participatory processes in order to prevent the data collected from failing to stimulate organizational change in working conditions.

## 1 Introduction

In recent years, there has been a noticeable rise in interest in how to contrast work-related stress (WRS) risks and promote well-being in academic settings, as several studies have shown an increase in stress symptoms among university staff (Johnson et al., 2019; Urbina-Garcia, 2020). Explanations for this worsening mental health situation point to the changes occurring in higher education institutions worldwide, such as a focus on internationalization, increased student numbers, and the growing importance of performance indicators to measure quality (Kinman and Johnson, 2019; Lee and Stensaker, 2021). Moreover, some scholars have highlighted that these adverse effects are a consequence of the spread of a neoliberal approach in universities. While these institutions have traditionally focused on education and societal betterment, this approach has emphasized high productivity and market-oriented strategies. This shift in mission has provoked emotional struggles due to the perceived dissonance between identity and academic work, negatively affecting well-being (Smith and Ulus, 2020).

Scholars agree that the essential condition for countering WRS risks and developing well-being at work is to design and implement a valuable methodology for assessing and managing risks related to workplace stress. To date, many models have been developed to effectively design this methodology (Leka et al., 2008; INAIL, 2018), but little is known about how the overall assessment and management pathway is put into practice in academia and what facilitates or hinders its progress (some exceptions: Pignata et al., 2018; Innstrand and Christensen, 2020). Filling this information shortage is critical for several reasons. Firstly, evidence from other business sectors suggests that practices adopted to manage this pathway play a critical role in well-being strategy effectiveness, as they influence the quality of data collected (Di Tecco et al., 2015). Secondly, Cox (2016) notes that although this pathway aims to identify, assess, and manage risks to employee health, in many contexts less attention is paid to how these risks are subsequently managed once they have been assessed. The risk management phase is often a “forgotten phase.” This has led to an implementation gap between assessment and intervention management. In academia, this gap can turn the stated emphasis on well-being into a rhetorical discourse, with data seldom used to inform management decisions. Moreover, neoliberal approaches can endorse an individualistic vision of well-being, focusing on personal responsibility in managing health and stress while overlooking underlying issues within the structures and programs of contemporary universities (Gill and Donaghue, 2016; Elraz and McCabe, 2023).

**Figure 1:**
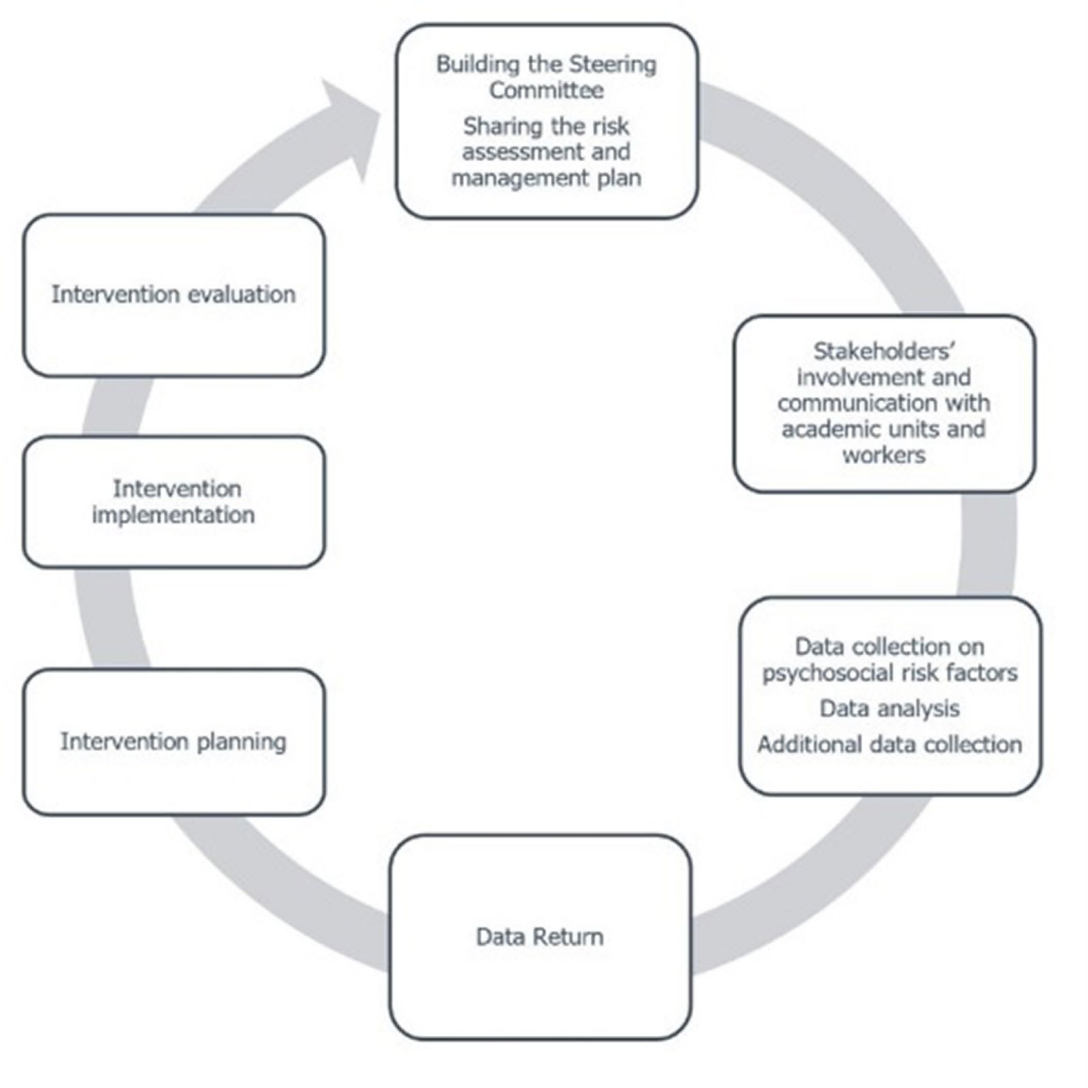
QoL@Work main phases for assessing and managing WRS risks.

Building on these premises, this perspective article argues that we need to consider the key enabling practices that could prevent the implementation gap in stress assessment-management pathways in academia. Understanding these practices could help mitigate the risk of well-being assessments becoming a mere rhetorical exercise rather than a catalyst for initiating organizational change. Therefore, this study aims to improve understanding of the factors that influence the realization of a WRS risk assessment-management pathway and how they may facilitate or hinder the transition from psychosocial risk assessment to the implementation of interventions. To this end, it explores the practices adopted for assessing and managing WRS risks in academia by referring to a group of Italian universities of the QoL@Work (Quality of Life at Work in academia) network. This network, established in 2016, involves 23 Italian universities and comprises academic work and organizational psychologists. One of the network’s missions is to share good practices for assessing and managing WRS risks in universities. The QoL@Work network has developed assessment tools (Brondino et al., 2022; Bruno et al., 2024) and a flexible and iterative model to evaluate and manage well-being and WRS risks (described in Ingusci, 2021). Based on previous models (Leka et al., 2008; INAIL, 2018; Innstrand and Christensen, 2020), it consists of defined phases, starting from the constitution of the group responsible for the process to the monitoring system of the improvement actions (see Figure 1).

## 2 Research design and methods

This perspective article is based on an empirical study that included all the universities of the QoL@Work network that carried out a WRS risk assessment and management pathway between 2016 and 2023. They were monitored in two steps.

The first step aimed to analyze the practices realized, from the assessment phase to intervention planning. Respondents were full and associate professors, and researchers in work and organizational psychology, members of the Qol@Work network, who participated in the WRS risk and well-being assessment and management pathway at their university. They were invited to complete a qualitative survey with open-ended questions. Questions included the description of each phase of the pathway, the organizational devices and tools used, as well as facilitators and barriers encountered (e.g., describe how the steering committee was formed, its goals and activities; describe the communication activities carried out, and the actors involved; describe which tools were used; describe any factors that hindered this phase; describe any factors that facilitated this phase). In cases of unclear or incomplete information, semi-structured telephonic interviews were conducted with the respondents to gain further insight into specific phases and the overall process of WRS risk assessment and management (e.g., could you please provide more details on the barriers of this phase?). In the second step, to monitor and follow up on the implementation phase (Figure 1), we engaged those respondents (i.e., the academic work and organizational psychologists who participated in the first step) whose universities had completed the assessment phase by at least one year and had moved to the intervention management phase. A questionnaire comprising both closed and open-ended questions was developed and made available online via the Qualtrics platform. Areas of inquiry included intervention design and implementation, their target population, the distribution of responsibilities, the allocation of resources, the duration of the process, the monitoring plan and methods, the practices of worker involvement, and the facilitators and barriers of the process (e.g., what types of interventions were designed? For what kind of reasons? Was the implementation plan formalized? Who was the target population of the interventions? Were the interventions implemented? Was a plan established to monitor and evaluate interventions? Which factors hindered the effectiveness of the implementation phase?).

In both steps of the study, informed consent for data usage was obtained from participants by providing a clear explanation in an email invitation, outlining the purpose of data collection, and assuring confidentiality and anonymity, according to the Helsinki Declaration.

All the data were anonymized, and qualitative data were analyzed using thematic analysis (Braun and Clarke, 2006; Bingham, 2023). First, following a deductive analysis process, topic codes were developed a priori in line with the purpose of the study and based on the QOL@work model for assessing and managing WRS risks (Figure 1). Topic codes were created for each phase, and data were sorted into categories related to practices and associated barriers and facilitators. After this step we inductively analyzed the data, defining codes. These codes were further refined through contrasts and comparisons. Finally, we reviewed code definition and labeling. We used traditional pencil and paper methods. Researchers independently analyzed the data and the credibility of the analysis, as a criterion for qualitative research, was assessed through supervision sessions to check the coding strategies and to review the interpretation of the data by discussing any reasons for variation (Barbour, 2001).

## 3 Results

In total, we collected data on 11 WRS risk assessment and management pathways carried out in 11 universities located in the northern, central, and southern regions of Italy. In the first step of the monitoring, 13 respondents were involved (for two universities, two key informants jointly completed the questionnaire), of whom 5 were additionally interviewed. Interviews lasted an average of 40 min (min: 20 – max: 50 min). In the second step, 7 of the 11 universities were involved, with 7 respondents completing the questionnaire.

Several key elements emerged by analyzing and comparing the practices adopted in the different phases of WRS risk assessment and management pathways. In particular, we focused on drivers and barriers to moving from assessing to implementing interventions (see Table 1). Due to space limitations, this study will not describe the specific planned and implemented interventions.

**Table 1:** Perceived facilitators and barriers to move from the assessment phase to the intervention management.

Phases	Facilitators	Barriers
Building the steering committee	Involving multiple actors Regular and formal meetings of the steering committee with academic governance Integration of technical and institutional roles	Responsibility for the process in only a few specialized and separate units Focus of the steering committee on the assessment phase rather than the process as a whole
Communication	Targeting specific academic units Making differentiated data understandable and actionable for different groups of employees Contextualizing the assessment process to interpret results Reaching precarious workers, who are less represented in institutional bodies Sustaining the perceived fairness of the process	The results of the assessment only communicated to the governance The results of the assessment only disseminated via digital technology Widespread and non-tailored dissemination of information Reports delivery without further discussion and involvement
Intervention planning	Focus-groups or workshops to engage employees in the action planning Clearly and formally identify the people responsible for implementing *each* action Strong governance endorsement for action implementation	Delayed return of data The assessment report is perceived as the endpoint of the process Unclear accountability of the transition from the assessment phase to the intervention phase

### 3.1 Building the steering committee

As Figure 1 illustrates, establishing the steering group^1^ and securing the commitment of academic governance to define and share goals, methods, and actions is the first phase of the WRS/well-being assessment and management pathway.

In this phase, differences were observed in the strategies adopted for constituting the steering group and in the interpretation of its role. Regarding its composition, in some universities, only one organizational unit (typically the health and safety office) was delegated to oversee the assessment phase and design the intervention plan. Regarding the steering group’s role, the assessment task was seen as the priority. Respondents underlined that great care was taken to design a reliable data collection method and procedure. To this end, experts in methodological issues (e.g., academic work and organizational psychologists) were involved and tasked with planning the assessment phase, analyzing the collected data, and reporting findings. In this case, some respondents noted the risk that delegating to a specialized but isolated organizational unit could result in the discharge of responsibility by other organizational units that are also critical to the well-being of the various components of the academic community.

Conversely, in other universities, several key organizational actors were involved, such as strategic roles within the administrative area (e.g., Human Resources and Communication Unit) and academic committees (e.g., members of the Guarantee Committee for Equal Opportunities, Employee Well-being, and Non-Discrimination at Work). In this context, the steering group members did not merely focus on the assessment: regular and formal meetings were held with academic governance. Moreover, other organizational actors who could provide insights into working conditions and contribute to the identification of context-specific indicators were consulted (e.g., trade unions, ethics committees, and confidential counselors for cases of discrimination, harassment, and mobbing).

“*We had formal meetings at each phase. Countless informal meetings for managing organizational aspects. Constant updates via email to the restricted group*” (University 1).

According to the respondents, these meetings facilitated the emergence of a shared belief that well-being is a challenge for the entire organization and that WRS risk assessment can become an organizational learning process. Integrating different institutional roles, especially when supported by academic governance, was seen as a choice that facilitated the collaboration across organizational units in data sharing and intervention planning.

### 3.2 Communication strategies: from information to building shared meanings

As depicted in Figure 1, communication plays a central role both before the data collection and after the assessment phase. Communication practices varied according to the targets and levels of involvement of academic working groups and stakeholders (administrative staff managers, department heads, and workers’ representatives). Three different patterns of communication practices were observed. In a first pattern, communication strategies mainly involved written communications targeted at academic governance. For example, reports were delivered and occasionally presented, but respondents worried that these reports might be archived without being utilized, especially when academic governance commitment was deemed lacking. In a second pattern, the communication strategy was characterized by activities addressed at the entire academic community, mainly mediated through computers: for example, professors, researchers, and administrative staff were informed about the WRS assessment by e-mail; after the data collection, wide dissemination of information to the entire academic community was realized by uploading the final report on the academic intranet, or by webinars. Despite the declared emphasis on worker involvement, some respondents pointed out that the utilization of such tools conveyed non-personalized communication. It did not help academic and non-academic staff understand the value of the WRS assessment. As a result, they were reluctant to complete the questionnaire. This reaction was observed particularly when well-being and psychosocial risk management were perceived as a low-priority issue and a concern for other professional categories but not for academics. A third pattern was less common. It was characterized by localized and tailored communication practices that were specifically targeted to academic divisions, sectors, and departments. These practices were considered pivotal by respondents because they reduced worker resistance to the assessment process and enabled outreach to precarious workers, such as research fellows, who are less represented in institutional bodies. In these cases, the goal was to ensure that the data could be understood and usable by different groups. Customized reports were prepared to provide each target group with the specific information they needed. Additionally, data return targeted to specific groups of workers or managers was considered an important strategy to ensure the perceived fairness of the process and to contextualize the interpretation of the results. The return of data with the goal of collaborative interpretation was perceived as a means of fostering a more robust connection between data and organizational processes.

*“Providing data with the aim of better interpreting it with feedback from stakeholders helps promote a better understanding of the processes being analyzed and reduces the risk of the evaluation being perceived as a judgment*” (University 3).

### 3.3 From assessment to intervention

As shown in Figure 1, the assessment phase is followed by identifying the improvement interventions to address critical issues and enhance organizational strengths. Indeed, addressing psychosocial risk factors requires an iterative cycle that includes intervention planning, implementation, and evaluation phases.

Most respondents reported a noticeable slowdown in the process and the perception that the implementation of the interventions lost priority. Several barriers emerged, including unclear accountability for the new phase, delayed data return long after the assessment, limited involvement of workers in the intervention design, and inadequate coordination among different organizational actors.

“*The process took a very long time, almost three years. We drafted a plan for future action, but it has not been implemented yet. For the faculty, it seems to be a bit of a taboo. In the meantime, with the pandemic, there has been a shift in prioritie*s” (University 4).

“*Once the assessment phase was over, the Health and Safety Office and Human Resources Department struggled to reach an agreement about how to define responsibilities.*” (University 5).

In some cases, interventions were not clearly identified, and the steering group ended its work without a clear handover. When the HR department was involved only at the end of the assessment phase, problems arose in agreeing on intervention priorities and deciding which organizational units were responsible for implementing specific actions.

Respondents mentioned strategies to mitigate the above barriers. First, from the beginning of the assessment phase, there should be an emphasis on fostering a culture of well-being management in which the assessment report is not seen as the endpoint of the process. Second, when designing interventions, simply providing a final report with suggested improvement actions may not be sufficient to initiate the implementation process. Conversely, in other cases, to support action implementation, some universities have involved employees not only in the data collection phase but also in the action planning phase through focus groups or workshops. A third element is to clearly and formally identify the people responsible for each action and involve the staff who could oversee its implementation, with strong endorsement from governance.

## 4 Discussion

This perspective article offers insights into critical factors that need to be considered to promote a process of assessing and managing academic WRS risks capable of reducing the ‘implementation gap’ and increasing the possibility of creating healthy academic conditions.

One relevant factor is how to set up and build the coordinating structure that supports the entire evaluation and intervention process. Two patterns of practice emerged from respondents’ experiences. The first pattern is characterized by a delegation-based approach: the management of the process is delegated to specific university offices specialized in safety and health, and to technical experts in WRS risk assessment. The main challenges perceived by the steering committee are related to methodological issues of ensuring reliable and valid data collection and analysis. The academic context is likely to reinforce this interpretation of the steering committee’s tasks because of the generally shared mission to produce high-quality research. Interestingly, our findings show that this pattern not only forgets the intervention phase, as suggested by Cox (2016), but also neglects the organizational aspects of the risk management process. Indeed, while great attention is paid to the methodological aspects of the assessment tasks, the steering committee tends not to attribute value to the construction of an organizational scaffolding that can support the translation of the collected data into interventions. A silo organizational culture seems to shape this pattern of practices. It can lead to negative consequences that contribute to the implementation gap: indeed, it creates barriers to information sharing and collaboration among organizational units that are empowered to implement the interventions to improve health conditions and well-being.

In contrast, a more integrative approach, resulting in a heterogeneous steering committee, is able to mitigate such barriers. It views the WRS risk assessment-management pathway primarily as an organizational change process, not just a methodology. Great value is placed on the time and effort required to create an inclusive coordinating structure in which diverse organizational actors can engage in dialogue and share a common vision of transforming working conditions to promote academic well-being. The involvement of key organizational actors responsible for initiatives in the intervention phase – such as HR managers – is a crucial task from the beginning of the pathway. This is consistent with previous studies (Suárez-Reyes and Van den Broucke, 2016) that have shown the importance of academic leaders and administrators considering well-being as a core issue across the university, rather than siloing it within a single unit. Within this pattern, the implementation gap can be overcome because the steering group is committed to creating the organizational conditions for ensuring the sustainability of the initiatives that emerge from the assessment phase by integrating them into existing academic strategic planning.

Based on our analysis, a second factor – which is connected to the first – may affect the implementation gap: it is related to the practices adopted to communicate and involve the academic community during the WRS risk assessment-management pathway. Indeed, the approach used in the first phase of the entire cycle (see Figure 1) is reflected in different logics of communication and worker engagement. More specifically, the delegation-based approach seems to be related to the first and second patterns of communication: vertical and dissemination. In these cases, as previously discussed, assessment is conceived as an objective and expert process based on the academic model which holds scholars responsible for generating scientific knowledge through rigorous research design and methods. The assumption is that the scientific framework may offer evidence-based solutions, and that vertical information or dissemination of knowledge is the only way to communicate with the stakeholders and produce change. However, this strategy could be perceived as distant from those who experience the working context daily, thus reinforcing the implementation gap.

In contrast, the integrative pattern used by the steering committee seems to sustain locally tailored practices of communication. The data return phase is considered as an opportunity to gain a deeper understanding of the context, by requiring a shift from an expert-oriented approach to a process consultation perspective (Schein, 1969). This pattern allows for the cultural adaptation of the entire process, through the integration of scientific knowledge and local knowledge of workers (Innstrand and Christensen, 2020; Ohadomere and Ogamba, 2021). In this direction, the WRS risk assessment and management pathway is conceived as a continuous learning process that requires participatory and personalized practices to address the unique working conditions of the specific academic population. Therefore, following this approach, it is a priority for academic governance to question and care about the quality of community involvement, also by reaching employees who are less represented. In line with previous studies, our results show that participatory practices need to be used in the WRS risk assessment-management pathway in order to reduce the implementation gap. First, investing in participatory practices can potentially counteract resistance and low organizational commitment to implementation. Active involvement of workers and key organizational stakeholders is crucial for both obtaining reliable findings and defining fitting and effective actions (Di Tecco et al., 2020). Secondly, participation is fundamental to promoting stakeholders’ empowerment, equipping them with the know-how and tools needed to embrace a healthy work environment (Innstrand and Christensen, 2020). In fact, the competencies of those managing the WRS risk assessment affect the process implementation (Di Tecco et al., 2015). Moreover, participatory practices resist the individualistic approach of the neoliberal model, which does not appear effective in addressing the root causes of WRS risks (Guthrie et al., 2017).

This study has some limitations: in most cases, data collection was retrospective to the WRS risk assessment, which may have influenced the richness of the data. Moreover, the findings are based on the representation of events as perceived by a single academic member, which could lead to a single-source bias. However, the respondents were able to observe and reflect back on the decisions and practices adopted during the process, because they were members or collaborators of the steering committee as work and organizational academic psychologists. Future research could benefit from including the multiple stakeholders involved in, or bypassed by, each university’s WRS risk assessment and management pathway. Analyzing their perspectives may enrich the understanding of the organizational dynamics affecting the implementation gap. Finally, future research needs to examine the relationship between the different patterns of practices in managing the stress risk assessment process in academia and the types of interventions implemented. Despite its limitations, this perspective article contributes to the academic health and well-being literature by highlighting how the WRS risk assessment and management pathway involves not only methodological issues but also organizational mindsets and choices. To minimize the risk of generating rigorous data without triggering organizational change in working conditions, academic governance should prioritize the creation of temporary organizational scaffolding to support participatory processes.

## Data availability statement

The raw data supporting the conclusions of this article will be made available by the authors, without undue reservation.

## Author contributions

AB: Writing – review & editing, Writing – original draft, Validation, Supervision, Methodology, Investigation, Data curation, Conceptualization. GD’A: Writing – review & editing, Writing – original draft, Validation, Supervision, Methodology, Investigation, Data curation, Conceptualization. SG: Writing – review & editing, Writing – original draft, Validation, Supervision, Methodology, Investigation, Data curation, Conceptualization.

## Footnotes

^1^According to the Italian guidelines (INAIL, 2018), based on the Italian Law on Health and Safety (81/2008), the Steering Group for the assessment and management of work-related risk must be formally established upon the Employer’s initiative and is generally made up of the Employer and/or delegated manager, the Health and Safety Manager, the Prevention and Protection Service Operator, the Occupational Physician (where appointed) and the workers’ representatives for Health and Safety.
